# Author Correction: Liquid crystal elastomer coatings with programmed response of surface profile

**DOI:** 10.1038/s41467-018-03634-w

**Published:** 2018-03-14

**Authors:** Greta Babakhanova, Taras Turiv, Yubing Guo, Matthew Hendrikx, Qi-Huo Wei, Albert P. H. J. Schenning, Dirk J. Broer, Oleg D. Lavrentovich

**Affiliations:** 1 Liquid Crystal Institute, Kent State University, Kent, OH 44242 USA; 2 Chemical Physics Interdisciplinary Program, Kent State University, Kent, OH 44242 USA; 3 Functional Organic Materials and Devices, Department of Chemical Engineering and Chemistry, Eindhoven University of Technology, 5612 AZAE Eindhoven, The Netherlands; 4 Institute for Complex Molecular Systems, Eindhoven University of Technology, P.O. Box 513, 5600 MB Eindhoven, The Netherlands; 5 Department of Physics, Kent State University, Kent, OH 44242 USA

Correction to: *Nature Communications* 10.1038/s41467-018-02895-9, published online 31 January 2018.

The original version of this Article contained errors in Figs. 1a, 2a, 3a, and 4b, in which the units on the scale bars incorrectly read ‘µm’ rather than the correct ‘nm.’ This has been corrected in both the PDF and HTML versions of the Article.

